# Supplementary material for: Mitochondrial ROS1 Increases Mitochondrial Fission and Respiration in Oral Squamous Cancer Carcinoma
Source: Cancers (Basel). 2020 Oct 1;12(10):2845. doi: 10.3390/cancers12102845 (PMC7599653; doi:10.3390/cancers12102845)
Supplement: Supplementary file 1 [file cancers-12-02845-s001.pdf]

cancers

Supplpementary Materials

Mitochondrial ROS1 Increases Mitochondrial Fission and respiration in oral squamous cancer carcinoma

Yu-Jung Chang, Kuan-Wei Chen and Linyi Chen

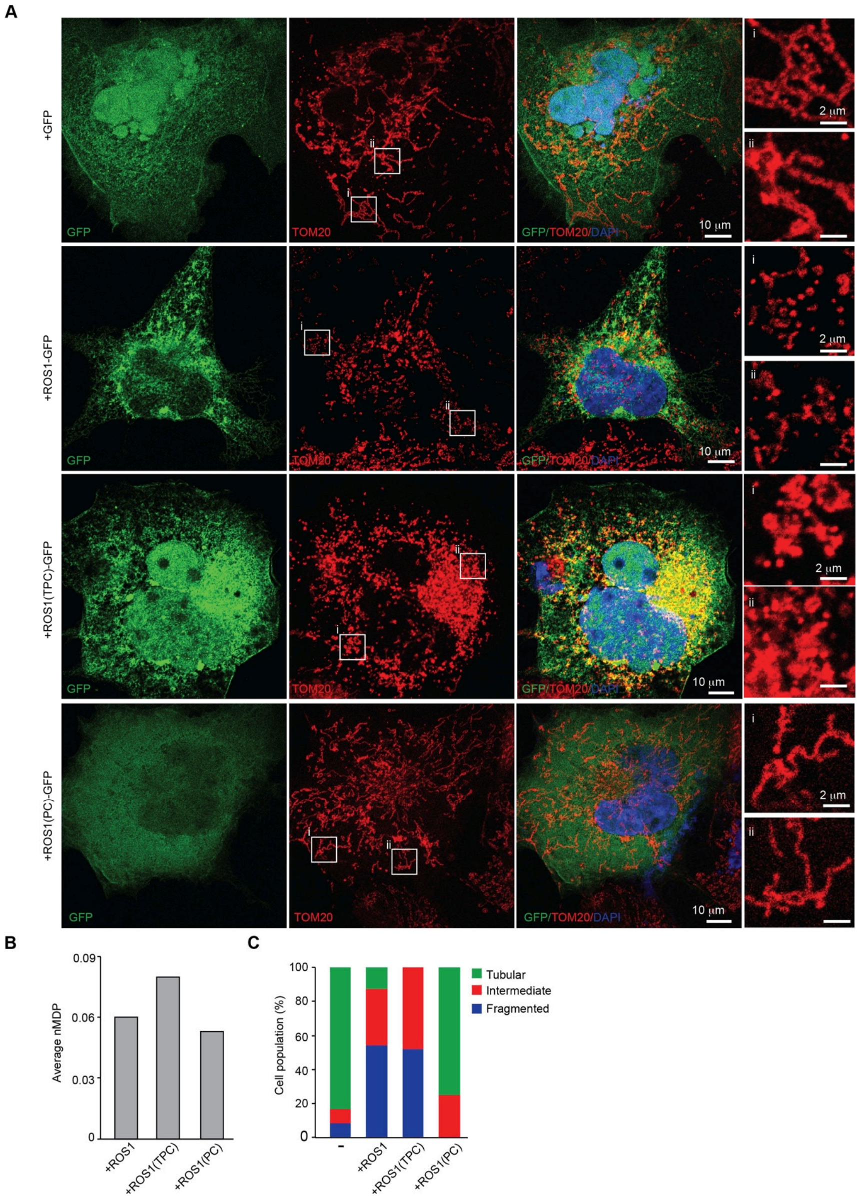

Figure S1. Distribution of ROS1 mutants and their effects on mitochondrial phenotype in COS7 cells. (**A**) COS7 cells were transfected with a vector expressing GFP, ROS1-GFP, ROS1(TPC)-GFP, or ROS1(PC)-GFP and then immunostained with anti-TOM20 (mitochondria, red) and DAPI (nucleus, blue). (**B**) Quantification of the average nMDP values. (**C**) Morphology of mitochondria from COS7 cells transfected with GFP (n = 9 cells), ROS1-GFP (n = 7 cells), ROS1(TPC)-GFP (n = 17 cells) or ROS1(PC)-GFP (n = 4 cells) was quantified. Scale bar: 10 μm (**A**); 2 μm (enlarged images).

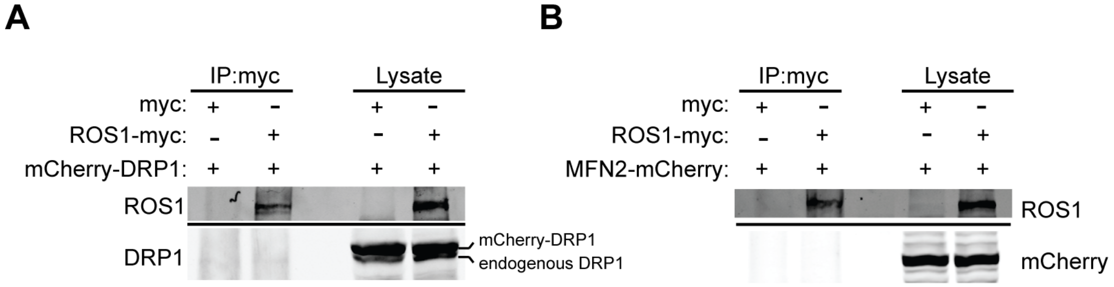

Figure S2. ROS1 oncoprotein does not interact with DRP1 or MFN2. (**A–B**) Lysates of 293T cells that had been transfected with myc vector or ROS1-myc along with mCherry-DRP1 (**A**) or MFN2-mCherry (**B**) were immunoprecipitated using anti-myc and immunoblotted for the indicated proteins.

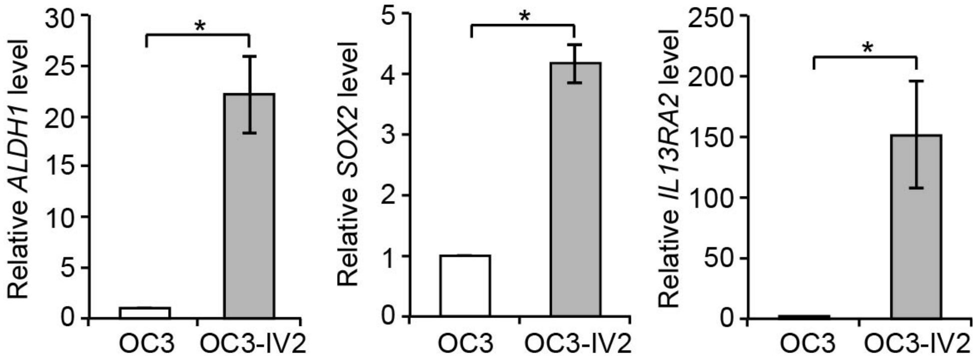

Figure S3. Comparison of stemness gene expression in OSCC cells. Relative expression of *ALDH1*, *SOX2*, and *IL13RA2* mRNAs in OC3 and OC3-IV2 cells was measured using Q-PCR. Data from three independent experiments are presented as mean ± SEM (*$p < 0.05$, paired Student's *t*-test).

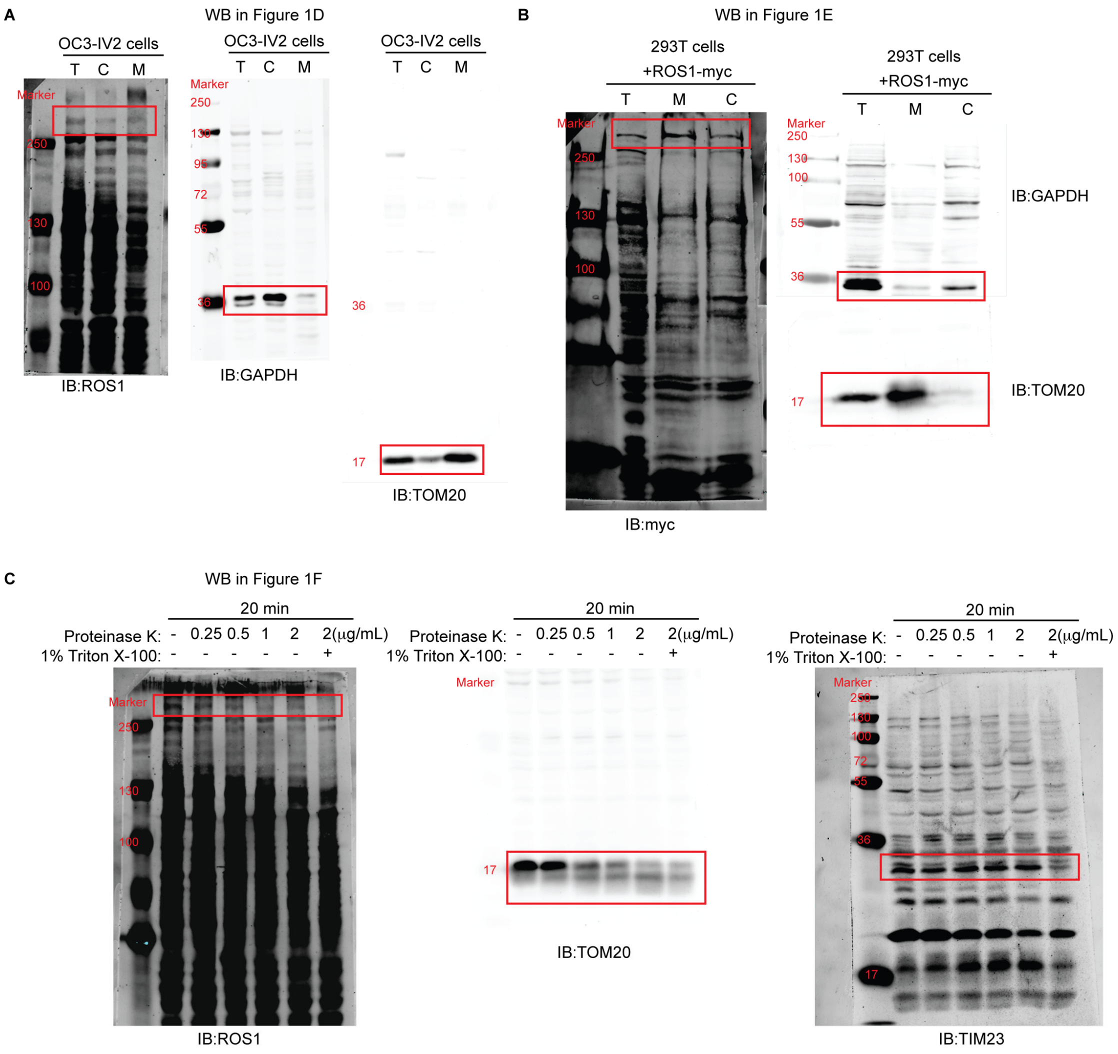

Figure S4. Full images of the western blot analysis for Figure 1.

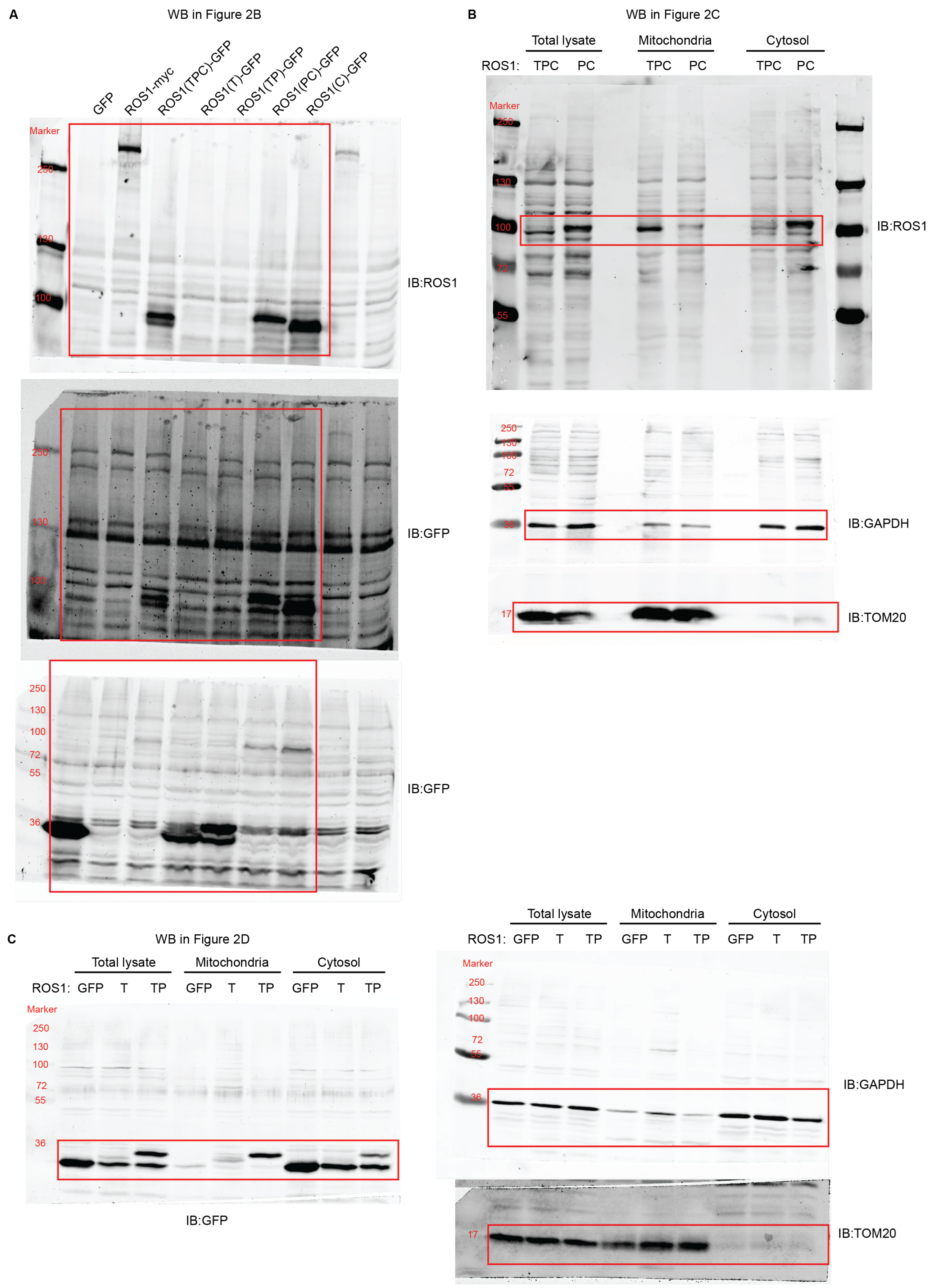

Figure S5. Full images of the western blot analysis for Figure 2.

© 2020 by the authors. Licensee MDPI, Basel, Switzerland. This article is an open access article distributed under the terms and conditions of the Creative Commons Attribution (CC BY) license (http://creativecommons.org/licenses/by/4.0/).
